# Effect and Mechanism of Modified Lichong Decoction on Inhibiting Proliferation and Inducing Apoptosis in AGS Gastric Cancer Cells

**DOI:** 10.1002/cnr2.70727

**Published:** 2026-11-11

**Authors:** Guoshan Shi, Liwei Ma, Hua Rong, Xu Li, Liping Wang, Shiyu Ni, Zhe Chen

**Affiliations:** ^1^ Qihuang College, Guizhou University of Traditional Chinese Medicine Guiyang Guizhou China; ^2^ Research Institute of Medicine & Pharmacy Qiqihaer Medical University Qiqihaer Heilongjiang China; ^3^ College of Pathology, Qiqihaer Medical University Qiqihaer Heilongjiang China; ^4^ Department of General Surgery The Fifth of Affiliated Hospital of Qiqihaer Medical University Daqing Heilongjiang China; ^5^ College of Public Health, Qiqihaer Medical University Qiqihaer Heilongjiang China

**Keywords:** apoptosis, gastric cancer, metabolomics, modified Lichong decoction, PI3k/Akt pathway

## Abstract

**Background:**

Lichong decoction, a classic Chinese herbal formula, has been applied to treat gynecological diseases and abdominal masses. According to pathogenesis of gastric cancer, we add some Chinese herbs to form the formula of modified Lichong decoction (MLCD) for treatment gastric carcinoma.

**Aim:**

To investigate the effect of MLCD on inhibiting proliferation and inducing apoptosis, and to explore its molecular mechanisms through metabolomics and pharmacological validation in AGS gastric cancer cells.

**Methods:**

The CCK‐8 assay, bromodeoxyuridine (BrdU) staining, morphological observation, DNA gel electrophoresis, and Annexin V‐FITC/PI staining were employed to evaluate the pharmacological effect of MLCD. The potential molecular mechanism of MLCD was evaluated by metabolomics and pharmacological verification.

**Results:**

The pharmacological effects showed that MLCD could inhibit proliferation, induce apoptosis, and affect the expression of apoptosis‐related genes and proteins (Bcl‐2, Bim, Bax, Fas, and FasL) in AGS cells. The metabolomics results verified that multiple signalling/metabolic pathways, including the PI3k/Akt signalling pathway, were involved in MLCD‐treated AGS cells. Subsequently, we conducted a pharmacological verification by combining with LY294002. The results showed that MLCD could reduce the protein expression levels of p‐Akt and p‐FoxO3a and enhance the nuclear translocation of FoxO3a. All results revealed that combining with LY294002 had more significant pharmacological effects. Meanwhile, the synthetical results of previous network pharmacology verified the related targets of MLCD for anti‐gastric cancer. The results showed that MLCD could reduce the gene and protein expression of P53, P21, LKB1, and AMPK in AGS cells.

**Conclusion:**

MLCD could suppress proliferation and induce apoptosis in AGS cells, which may involve the mitochondrial apoptotic pathway, death receptor pathway, P53/P21 pathway, LKB1/AMPK pathway, and PI3k/Akt pathway.

## 1 Introduction

According to Global Cancer Watch statistics for 2022, gastric cancer accounts for 4.9% of newly diagnosed cases globally, with a mortality rate of 6.8% [1]. In China, the incidence and mortality of gastric carcinoma rank third among all malignancies [2]. The primary treatment strategy for gastric cancer is surgical resection [3]. Patients with gastric carcinoma are often diagnosed at an advanced stage, with a five‐year survival rate of less than 30% [4]. Although advances in surgical methods, radiotherapy, chemotherapy and neoadjuvant therapy have remarkably improved the survival rate of patients with gastric carcinoma, the prognosis for those with advanced gastric carcinoma remains disappointing due to poor outcomes and limited treatment options. Therefore, new therapeutic approaches are needed [5]. Chinese herbs, characterised by multiple targets and fewer adverse reactions, offer significant advantages in improving the quality of life for gastric cancer patients. Natural medicines are also a rich source for treating diseases and developing synthetic drugs [6].

Lichong decoction, a classic herbal formula, was used to treat gynaecological diseases and abdominal masses, written by Zhang Xichun, a famous doctor in the Qing Dynasty and recorded in ‘Intergrating Chinese and Western Medicine’, composed of 9 traditional Chinese herbs (*Astragalus membranaceus* (Fisch.) Bge., *Codonopsis pilosula* (Franch.) Nannf., *Atractylodes macrocephala* Koidz., *Dioscorea opposita* Thunb., *Dioscorea opposita* Thunb., *Anemarrhena asphodeloddes* Bge, *Sparganium stoloniferum* Buch.‐Ham., Curcuma phaeocaulis Val. and Galli Gigerii Endothelium Corneum) [7]. The deficiency of Lichong decoction in the treatment of gastric cancer results in an insufficient pharmaceutical effect on the supplementation of primordial Qi and elimination of mass. According to the principle of syndrome differentiation to treat tumours in traditional Chinese medicine (TCM), combining with pathogenesis of gastric cancer which were deficiency of primordial Qi, accumulation of pathogenic toxins in the body and retention of phlegm, fluid and stasisloodhinders. Lichong decoction subtracts *Dioscorea opposita* Thunb., *Trichosanthes kirilowii* Maxim. and *Anemarrhena asphodeloddes* Bge, which are medicines for clearing heat and supplementing Yin, *Codonopsis pilosula* (Franch.) Nannf., which is medicine for supplementing of stomach Qi and Galli Gigerii Endothelium Corneum which is medicine for aid digestion and eliminate food accumulation, adds *Oldenlandia diffusa* (Willd.) Roxb., *Scutellaria barbata* D. Don, which are antitoxicant medicines for clearing heat, *Hirudo nipponica* Whitman, *Sargassum fusiforme* (Harv.) Setch., *Gekko swinhonis* Guenther, which are resolving masses and *Panax quinquefolium* L., *Cervus elaphus* Linnaeus, *Chinemys reevesii* (Gray), which are supplementing of primordial Qi, to form a formula of modified Lichong decoction (MLCD).

A reflux extraction method, as described in the literature [8], was employed to prepare the compound prescription. In previous studies [9, 10], we confirmed that a co‐extract of alcohol and water from modified Lichong decoction (MLCD) exhibited stronger anti‐proliferation effects in gastric cancer cells than either the alcohol or water extract alone. This co‐extract also had the most significant inhibitory effect on the proliferation of AGS cells among multiple gastric cancer cell lines (MNK‐45, AGS, MGC‐803, SGC‐7901 and BGC‐823) and induced apoptosis in AGS cells while exerting a weaker inhibitory effect on GES‐1 cells. In summary, AGS gastric cancer cells were selected as the experimental cells and the specific effects and molecular mechanisms of MLCD were further explored in this study.

## 2 Materials and Methods

### 2.1 Drugs and Reagents

FBS (cat. no. SV30087.02) was acquired from Hyclone (USA); DMEM culture medium (cat. no. GUMD‐B301) was acquired from HyCyte (China). CCK‐8 (cat. no. CK04) was purchased from Dojindo Laboratories (Japan); FITC BrdU kit (cat. no. 559619) and Annexin V‐FITC/PI Staining kit (cat. no. 556547) were acquired from BD Biological Company (USA). TB Green Premix Ex Taq (cat. no. RR420A), Prime Script RT reagent Kit (cat. no. RR037A) and DNA extraction kit (cat. no. 9765) were purchased from TaKaRa Corporation (Japan). TUNEL reagent kit (cat. no. c10618) was purchased from Thermo Fisher Scientific Company (USA). All antibodies were obtained from Abcam Biological Company or CST Biological Company (USA).

Human AGS cell was acquired from Shanghai Institute of Life Sciences (China). The AGS cell was cultured with DMEM culture medium and incubated in a thermotank for 37°C with 5% CO_2_. After approximately 1–2 days, AGS cells were used to execute the experiment when they overgrew the bottom of the petri dish.

### 2.2 Experimental Drug

All Chinese herbs were acquired from Guizhou Yipin Co. LTD. (Guizhou Province, China) and authenticated by Prof. Yu Sun from the Natural Pharmaceutical Chemistry Center of Qiqihar Medical University [9]. All of the above plant names have been checked at http://mpns.kew.org. MLCD was prepared by the method of previous literature [9]. Briefly as follows. The total mass of the 12 Chinese herbs was 240 g. The herbs were drenched and extracted by using 85% ethanol at a liquid‐to‐solid ratio of 8:1 for 1.5 h each time, repeated three times and then distilled water at a liquid‐to‐solid ratio of 10:1 for 1 h each time, repeated three times. The blended extracts were concentrated by using a rotary evaporator (Buchi, R‐220, Switzerland) and then dried by using a Vacuum Pump (Huxi analytical Instrument Factory, SHZ‐3, China) under low pressure to acquire MLCD (lyophilized powder). According to the mass fraction, MLCD was prepared at 150, 300 and 600 μg/mL as the low, medium and high dose groups by using a DMEM culture medium.

### 2.3 CCK‐8 Assay

AGS cells (2 × 10^4^) were seeded into 96‐well microplates containing 200 μL of DMEM culture medium and exposed to MLCD (150, 300, 600 μg/mL). Meanwhile, a blank control group without cells was set up. At the end of each period, 20 μL of CCK‐8 reagent was added to the microplate. The cells exposed to MLCD were then incubated in a thermotank for 3 h. Finally, optical density (OD) values were detected at 450 nm using a multifunctional Reader (TECAN, Austria). The mean value from five wells in different groups was acquired. To further verify that PI3k/Akt transduction pathways participated in regulating cellular proliferation, the cells were subjected to MLCD combined with LY294002 (40 μM LY294002 was added 1.5 h before the MLCD reaction concluded). The cell growth inhibition rate was calculated using the equation [1 −OD (treatment group)/OD (control group)].

### 2.4 Bromodeoxyuridine (BrdU) Test

AGS (1 × 10^5^) cells were seeded into 24‐well confocal microplates (black walls and transparent bottom) containing 1 mL of DMEM culture medium and exposed to MLCD (150, 300, 600 μg/mL) for 48 h. The survival rate of AGS cells was determined using a BrdU kit according to the manufacturer's instructions. The following reagents were sequentially added to each well of the microplate: 100 μL of BrdU solution at room temperature for 25 min, 100 μL of fixed reagent at room temperature for 25 min, 100 μL of Triton X‐100 at room temperature for 25 min, 100 μL of DNase reagent at 37°C for 30 min and fluorescent antibody or DAPI solution at 37°C in the dark for 30 min. After each step, the wells were washed with a buffered solution. Finally, photographs were acquired using a laser microscope (Zeiss, LSM710, Germany) at the same laser intensity. The fluorescent intensity of the stained cells in the visual field was counted to represent surviving cells.

### 2.5 Analysis of Morphological Changes

AGS (2 × 10^5^) cells were seeded into 6‐well microplates or confocal microplates (black wall and bottom transparent) containing 1 mL of DMEM culture medium and exposed to MLCD (150, 300, 600 μg/mL) for 48 h. Morphological changes were observed and photographed using an optical microscope (Zeiss Company, ObserverA1, Germany).

According to the TUNEL kit instructions, TdT buffer was added to the wells of 6‐well confocal microplates and incubated at 37°C for 10 min. Subsequently, the TdT reaction solution was added and the reaction was incubated at 37°C for 60 min. Following this, 3% BSA and 0.1% Triton X‐100 were added and the mixture was incubated at 37°C for 5 min. The TUNEL staining solution was then added and incubated at 37°C for 30 min. Finally, quenching gel was added to the wells and changes in the fluorescence intensity of treated cells were observed and photographed using a laser confocal microscope (Zeiss Company, LSM710, Germany) with consistent laser intensity.

Cells treated with MLCD were collected in EP tubes. The ultrastructural changes of cells were examined and photographed using a Transmission Electron Microscope (TEM) (Hitachi Limited, HT7700, Japan). Subsequent procedures, such as fixation, slicing and staining, were previously described in [11].

### 2.6 Apoptosis of Analysis

AGS cells were seeded and treated as described above. DNA laddering in cells was detected by agarose gel electrophoresis. According to the DNA extraction kit instructions, cells were collected into EP tubes and RNaseA, proteinase K and lysis buffer were added. The tubes were then placed in a 70°C water bath for 5 min, followed by the addition of anhydrous ethanol to precipitate the DNA. The DNA was transferred into a DNA purification column, eluted and obtained by centrifugation at 12 000 × *g* for 10 min. The DNA samples from each group were loaded onto an agarose gel for electrophoresis and images were observed under an imaging system.

AGS cells were seeded and treated as described above. The rate of apoptosis was detected using Annexin V‐FITC/PI staining. After 48 h of treatment with MLCD, cells were collected and rinsed once with cold PBS, resuspended in 300 μL of buffer and then stained with 15 μL of Annexin V‐FITC and 10 μL of PI. The stained cells were gently resuspended and incubated for 10 min at 37°C in a luciferase environment. To further verify that the PI3k/Akt pathway is involved in regulating apoptosis, AGS cells were treated with MLCD combined with LY294002. The status of the stained cells was evaluated by flow cytometry (BD, FACSCalibur, USA).

### 2.7 Metabolomics Analysis

AGS cells treated with MLCD 0 μg/mL (Control) groups or 600 μg/mL (MLCD‐H) groups chosen for metabolomics. One mL of cooled extraction solution (methanol/acetonitrile/H_2_O) was blended with the sample and vortexed to precipitate protein. The mixture was then centrifuged at 12 500 *g* for 35 min at 4°C. The same method was used to prepare quality control (QC) samples. LC–MS detection and data analysis were performed using METWARE corporation (China). Metabolomics were analysed using a quadrupole time‐of‐flight mass spectrometer (Sciex Triple TOF 6600) coupled to hydrophilic interaction chromatography via electrospray ionisation at Shanghai Applied Protein Technology Co. Ltd. LC separation was performed on an ACQUITY UPLC BEH Amide column (2.1 mm × 100 mm × 1.7 μm, waters) using a gradient of solvent A (25 mM ammonium acetate and 25 mM ammonium hydroxide in water) and solvent B (acetonitrile). The condition of gradient was shown in Table 1. The condition of Mass spectrum was shown in Table 2. The orthogonal partial least‐square discriminant analysis (OPLS‐DA) was established to assess metabolic variation in AGS cells. Then, the sevenfold cross‐validation and response permutation testing were used to evaluate the robustness of the model. The variable importance in the projection (VIP) value of each variable in the OPLS‐DA model was calculated to indicate its contribution to the classification. Significance was determined using an unpaired Student's *t*‐test. VIP value > 1 and *p* < 0.05 was considered as statistically significant. Furthermore, the analysis of metabolic pathway were performed using MetaboAnalyst 5.0 software. To further explore the impact of differentially expressed metabolites, enrichment analysis was performed. KEGG pathway enrichment analyses were applied based on the Fisher’ exact test, considering the whole metabolites of each pathway as background dataset. And only pathways with *p*‐values under a threshold of 0.05 were considered as significant changed pathways.

**TABLE 1:** The condition of mobile phase.

Time (min)	The gradient of solvent A and solvent B
0.0	95% A and 5% B
2.0	80% A and 20% B
5.0	40% A and 60% B
6.0	1% A and 99% B
7.5	1% A and 99% B
7.6	95% A and 5% B
10.0	95% A and 5% B

**TABLE 2:** The condition of mass spectrum.

Facility	ESI+	ESI−
Spray voltage	3500	3200
Sheath gas (Arb)	30	30
Aux gas (Arb)	5	5
Ion transfer tube temperature	320	320
Vaporizer temperature	300	300
Scan range	75–1000	75–1000
Resolution	35 000	35 000
AGC target	1 × 10^6^	1 × 10^6^
RF lens (%)	50	50
Scan range	75–1000	75–1000

### 2.8 Detection of the Expression Levels of Related Genes via qRT‐PCR

A qRT‐PCR assay was employed to analyse the expression levels of related genes. Briefly, AGS cells were seeded and treated as described above. After 48 h of treatment with MLCD or MLCD combined with LY294002, cells were collected with Trizol reagent. Total RNA from cells was obtained using the phenol‐chloroform method. The purity and concentration of RNA were assessed using a spectrophotometer at *A*_260nm_/*A*_280nm_. The total RNA was reverse‐transcribed into cDNA according to the manufacturer's instructions. The obtained cDNA was used to quantify the target genes using a PCR instrument (ABI, Q5, USA) with the TB Green Premix ExTaq kit. The cycling conditions for the target genes were as follows: 95°C for 30 s, followed by 40 cycles of denaturation at 95°C for 5 s and annealing and extension at 65°C for 32 s. The message of primers was as Table 3. The expression levels of related genes were normalised against β‐actin. The fold change in gene expression was counted using the 2^−△△Ct^ method.

**TABLE 3:** Sequence and length of PCR primers.

Gene	Sequence	Length (bp)
β‐Actin	Forward: 5′‐GTTGTCGACGACGAGCG‐3′ Reverse: 5′‐GCACAGAGCCTCGCCTT‐3′	93
Bcl‐2	Forward: 5′‐GAGAAATCAAACAGAG GCCG‐3′ Reverse: 5′‐CTGAGTACCTGAACCGGCA‐3′	106
Bax	Forward: 5′‐AG CTTCTTGGTGGACGCAT‐3′ Reverse: 5′‐CAGAGGCGGGGTTTCATC‐3′	101
Bim	Forward: 5′‐GATAGTGGTTGAAGGCCTGG‐3′ Reverse: 5′‐CCTCCCTACAGACAGAGCCA‐3′	102
Fas	Forward: 5′‐GCATCTGGACCCTCCTACCT‐3′ Reverse: 5′‐TCCTCAATTCCAATCCCTTG‐3′	108
FasL	Forward: 5′‐GCACAC AGCATCATCTTTGG‐3′ Reverse: 5′‐GGACCTTGAGTTGGACTTGC‐3′	109
P53	Forward: 5′‐GCTCGACGCTAGGATCTGAC‐3′ Reverse: 5′‐GCTTTCCACGACGGTGAC‐3′	96
P21	Forward: 5′‐CATGGGTTCTGACGGACAT‐3′ Reverse: 5′‐AGTCAGTTCCTTGTGGAGCC‐3′	107
Foxo3a	Forward: 5′‐AGTTCCCTCATTCTGGACCC‐3′ Reverse: 5′‐CTTCAAGGATAAGGGCGACA‐3′	102
*LKB1*	Forward: 5′‐CGAGGACCTCTTCGACATCG‐3′ Reverse: 5′‐TGTCCATTGTGACTGGCCTC‐3′	96
*AMPK*	Forward: 5′‐CACCAGGATCCTTTGGCAGT‐3′ Reverse: 5′‐AGAATCAGGTGGCTTGTCG‐3′	102

### 2.9 Detection of the Expression Levels of Related Proteins via Western Blotting

A Western blotting assay was used to analyse the expression levels of related proteins. Briefly, cells were seeded into a 100‐mm foster vessel and exposed to MLCD or MLCD combined with LY294002 for 48 h and lysed using lysis solution containing 1 mM protease and phosphatase inhibitors in an ice bath for 0.5 h. The suspension was obtained by centrifugation at 12000 × g for 20 min and protein concentrations were detected using a BCA assay Kit. Overall, 20 μL of proteins was isolated via polyacrylamide gel electrophoresis and transferred to a PVDF membrane. The membrane was blocked with BSA reagent at room temperature for 2 h, probed with primary antibodies from CST or Abcam overnight at 4°C, incubated with an anti‐rabbit/mouse‐IgG for 2.0 h at 37°C and then exposed using a gel image system (Bio‐Rad, ChemiDoc MP, USA). The blotting intensity was quantified using the ImageLab. The proteins levels are represented as optical densities. The expression of targeted proteins was normalised against GAPDH.

### 2.10 Analysis of the Nuclear Translocation of the FoxO3a Protein

The immunofluorescence test was employed to analyse the nuclear translocation of FoxO3a protein using a confocal laser microscopy (Zeiss, LSM710, Germany). AGS cells treated with MLCD or MLCD combined with LY294002 were seeded into a 6‐well confocal microplate. Each well was managed in sequence as follows: paraformaldehyde fixation at 37°C for 20 min, TritonX‐100 to permeabilisation at 37°C for 20 min, PBS buffer containing 1% BSA blocking at room temperature for 1.5 h, diluted FoxO3a antibody (Cat No. ab47285, 1:300) incubation overnight at 4°C, diluted fluorescent secondary antibody staining for FoxO3a protein at 37°C for 1 h in the dark and DAPI reagent staining of the nucleus at room temperature for 15 min. Images were obtained using confocal laser microscopy at the same laser intensity.

### 2.11 Statistical Analysis

Data are shown as the means value ± SD from at least three independent tests. The means of different groups were compared using ANOVA. Statistical analyses were executed using SPSS18.0 software. Distinction was ascertained according to statistical significance when *p < 0.05, **p < 0.01 and ***p < 0.001.

## 3 Results

### 3.1 MLCD Suppression of AGS Cells Viability

Literature indicates that uncontrolled cell multiplication is a property of tumour cells. Hence, inhibiting cell multiplication may serve as the main target for screening and developing anti‐tumour medicine [12]. In this study, cellular proliferation was detected via a CCK‐8 test. AGS cells were treated with 0, 150, 300 and 600 μg/mL of MLCD for 24, 48 or 72 h. These data show that MLCD inhibited cellular multiplication in a dose‐dependent manner (Table 4).

**TABLE 4:** Cellular proliferation of AGS cells exposed to MLCD (0, 150, 300 and 600 μg/mL) at different time points (24, 48 or 72 h).

Groups (μg/mL)	24 h		48 h		72 h	
	OD	Inhibition rate %	OD	Inhibition rate %	OD	Inhibition rate %
0	2.016 ± 0.094	—	2.435 ± 0.104	—	2.872 ± 0.089	—
150	1.673 ± 0.102*	17.03	1.549 ± 0.106**	36.38	1.665 ± 0.096***	42.03
300	1.340 ± 0.092**	33.55	1.195 ± 0.086***	50.90	1.135 ± 0.076***	60.48
600	0.999 ± 0.082***	50.43	0.693 ± 0.071***	71.52	0.669 ± 0.086***	76.71

*Note:* Data is shown as a mean ± SD from independent triplicate experiments. Compared with the control (0 μg/mL) group.

^*^ *p* < 0.05.

** *p* < 0.01.

*** *p* < 0.001.

### 3.2 Effects of MLCD on the Survival Rate in AGS Cells

Microscopic observation showed that the number of cells labelled with BrdU was higher in the control group due to a greater number of living cells, whereas the number of labelled cells gradually decreased with the increasing concentration in the MLCD treatment group (Figure 1A). The results for the survival rate in MCLD treatment groups were statistically significant when compared with the control group (Figure 1B).

**FIGURE 1:**
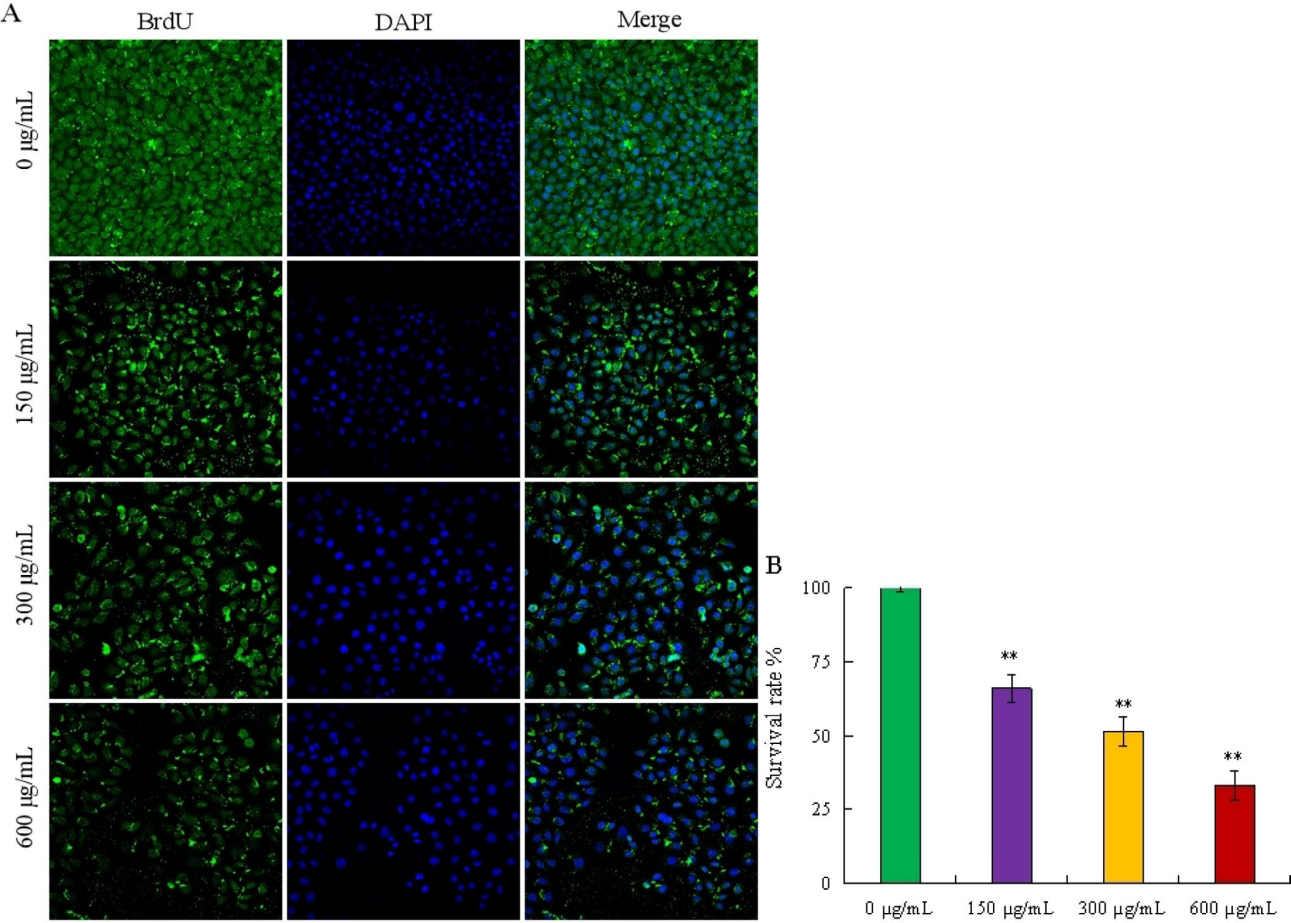
The survival rate of AGS cells exposed to MLCD (0, 150, 300 and 600 μg/mL) for 48 h. (A) A representation of the BrdU staining. (B) A histogram showing the differences between groups. Data are presented as the means ± SD from independent triplicate experiments. **p < 0.01, compared with the control (0 μM) group.

### 3.3 Effects of MLCD on Morphological Changes of Apoptosis in AGS Cells

The prohibitive activity on cell multiplication can be realised by inducing apoptosis [13]. In this study, the AGS cells in the control group grew densely, were round and had good transparency. In the groups treated with MLCD, as the dose of MLCD increased, the cells gradually exhibited morphological characteristics, such as a decrease in number, weakened adhesion, the appearance of irregular corners and a small amount of cell debris (Figure 2A).

**FIGURE 2:**
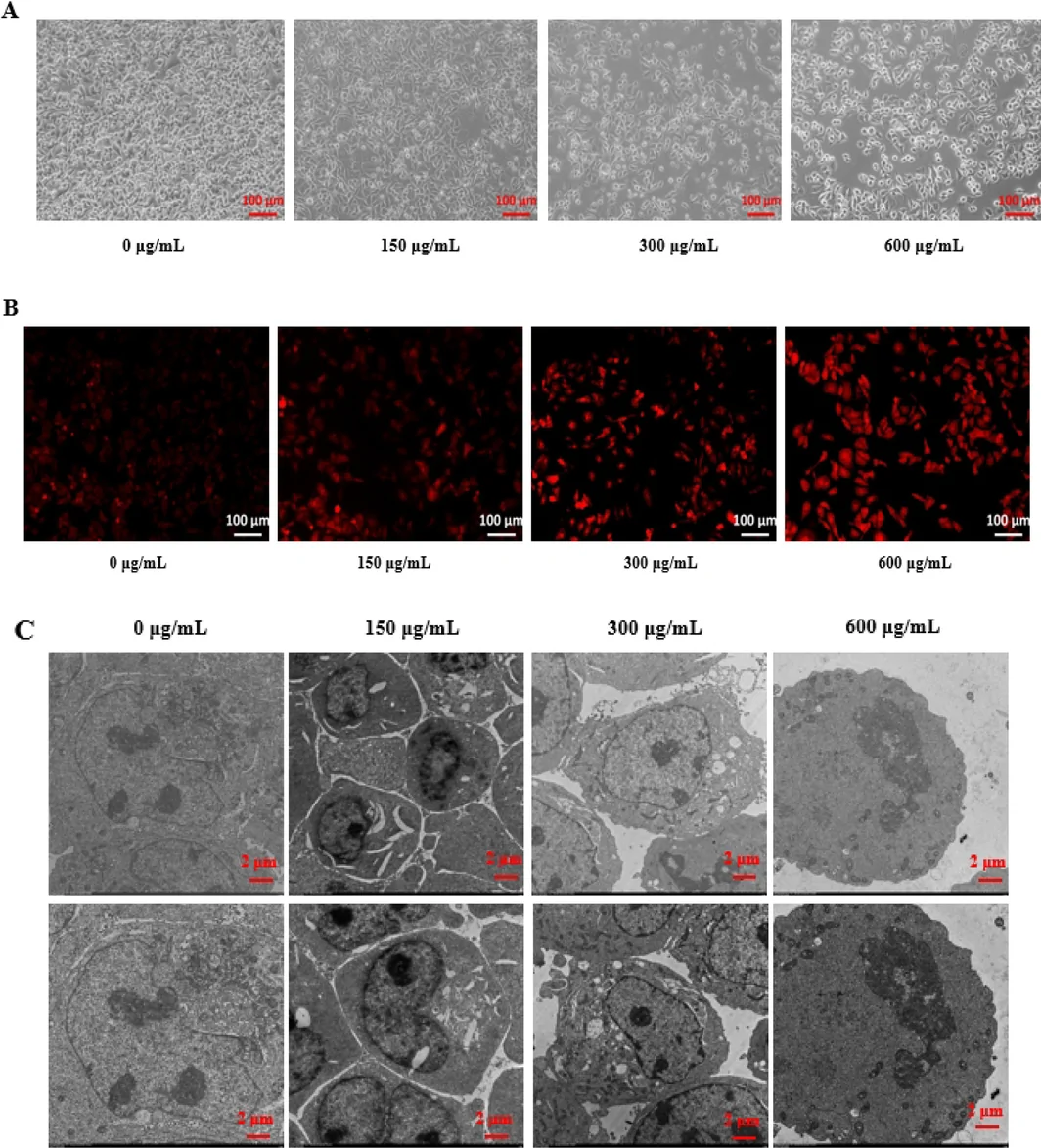
The morphological changes of apoptosis in AGS cells exposed to MLCD (0, 150, 300 and 600 μg/mL) for 48 h. (A) A representation of the morphological changes observed under an optical microscope. (B) A representation of the TUNEL staining observed under a laser confocal microscope. (C) A representation of the ultrastructural changes observed under a transmission electron microscope.

In the TUNEL staining experiment, cells in the control group grew normally; TUNEL‐stained cells showed weak red fluorescence under the confocal microscope. In the groups treated with MLCD, as the dose of MLCD increased, the number of TUNEL‐stained cells increased, indicating an increase in apoptotic cells and the fluorescence intensity of these cells also increased significantly (Figure 2B).

TEM observation showed that, in the control group, AGS cells exhibited marked nuclear pleomorphism, a high nucleocytoplasmic ratio and abundant organelles. With the increase in MLCD concentration, cells treated with MLCD exhibited morphological features of apoptosis, such as nuclear condensation, vacuolar degeneration of organelles, widened perinuclear space and abundant cytoplasmic lipid accumulation (Figure 2C).

### 3.4 Effects of MLCD on Apoptosis in AGS Cells

During apoptosis, DNA cleavage is an important indicator of this irreversible process of cell death. Anti‐tumour drugs can induce DNA double‐stranded damage in tumour cells, thereby promoting apoptosis [14]. The results of DNA agarose gel electrophoresis show a uniform band in the control group and that, as the MLCD concentration increased, bands of a typical ladder pattern were presented in the groups treated with MLCD (Figure 3A).

**FIGURE 3:**
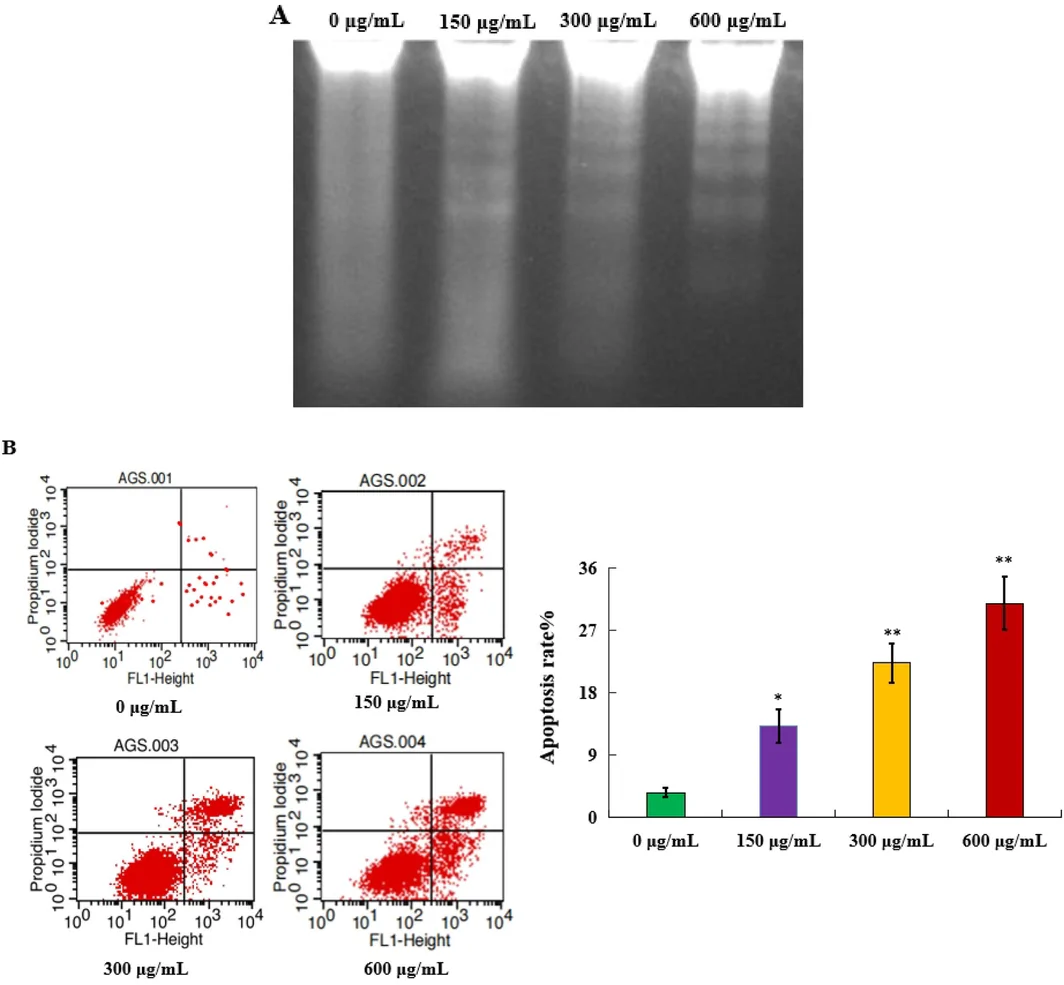
The apoptosis of AGS cells exposed to MLCD (0, 150, 300 and 600 μg/mL) for 48 h. (A) A representation of DNA agarose gel electrophoresis. (B) The apoptotic rate was detected by flow cytometer with Annexin‐FITC/PI dyeing. Data are presented as the means ± SD from independent triplicate experiments. *p < 0.05, **p < 0.01, compared with the control (0 μM) group.

The results of apoptotic rate showed that compared with the control group (3.58% ± 0.69%), the total apoptotic rates were gradually increased from (13.27% ± 2.40%) to (22.28% ± 2.84%) and (30.89% ± 3.83%) when MLCD was set at 150, 300 and 600 μg/mL for 48 h (Figure 3B).

### 3.5 Expression Levels of MLCD‐Induced Apoptosis‐Related Genes/Proteins in AGS Cells

Studies confirmed that both traditional cytotoxic medicines and targeted curative medicines have anti‐cancer effects by inducing apoptosis [15]. Meanwhile, during the process of apoptosis, there are two different mechanisms, namely the mitochondrial apoptotic pathway and the death‐receptor pathway [16]. The mitochondrial pathway is regulated by Bcl‐2 family proteins, which include pro‐apoptotic proteins (including Bax, Bim, etc.) and anti‐apoptotic proteins (such as Bcl‐2, Bcl‐XL, etc.) [17, 18]. The Fas/FasL signalling pathway is one of the classical death‐receptor‐regulated pathways in tumour cells. In this pathway, Fas interacts with its ligand FasL to regulate apoptosis and participates in the physiological and pathological processes of cells [19, 20]. In this study, we evaluated the expression levels of apoptosis‐related genes in AGS cells induced with MLCD for 48 h. The results exhibited that, compared with the control group, the mRNA and protein levels of Bcl‐2 progressively reduced and the mRNA and protein levels of Bax, Bim, Fas and FasL successively augmented with increasing MLCD concentration (Figure 4).

**FIGURE 4:**
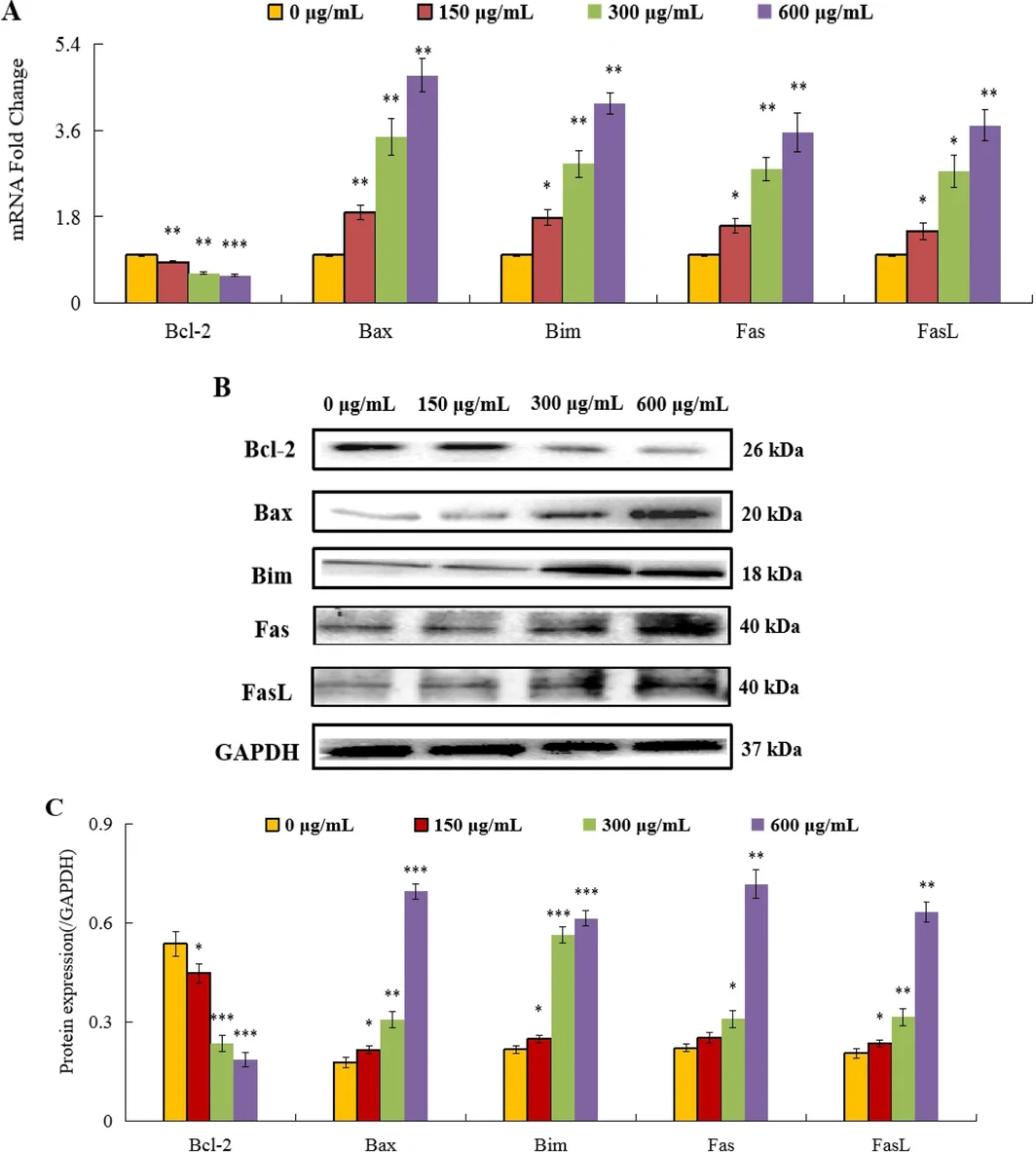
The expression results of apoptosis‐related genes and proteins were shown in AGS cells exposed to MLCD (0, 150, 300 and 600 μg/mL) for 48 h. (A) The results of genes expression. A histogram showing the differences between groups. (B) A representation of proteins expression. (C) A histogram showing the differences between groups. All data are presented as the means ± SD from independent triplicate experiments. *p < 0.05, **p < 0.01, ***p < 0.001, compared with the control (0 μM) group.

### 3.6 Metabolomics Analysis Results

In the test, untargeted metabolomic profiles were performed in AGS cells treated with Control and MLCD‐H, based on LC–MS/MS. The pearson correlation of QC samples and PCA (Principal Component Analysis) of score plot were visualized in Figure 5A,B, which demonstrated that the detective process was stable and the detective data were reliable. Moreover, a 200‐permutation test was performed to confirm the reliability of the Orthogonal partial least squares discrimination analysis (OPLS‐DA) models and significant difference of metabolites was emerged by the diagram of OPLS‐DA S‐plot (Figure 5C).

**FIGURE 5:**
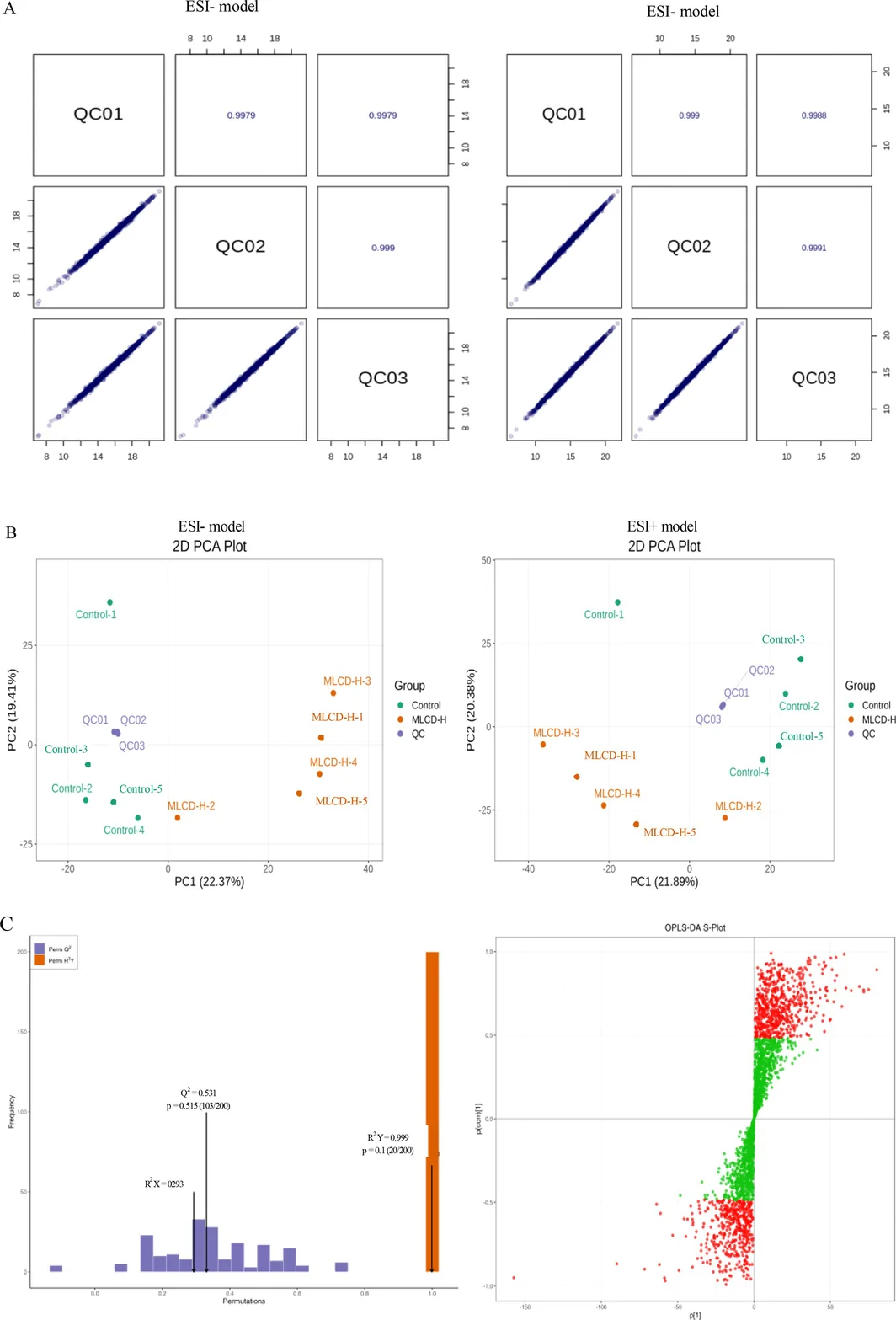
QC samples test and OPLS‐DA of control and MLCD‐H group. (A) The Pearson correlation of QC samples. (B) PCA of score plot. (C) OPLS‐DA permutation test and the diagram of OPLS‐DA S‐plot.

Compared with the control group, ninety‐five compounds (56 up‐regulation and 39 down‐regulation) were defined as differential metabolites according to ANOVA with *p* < 0.05, FC ≥ 1.2 or VIP ≥ 1, including adenosine monophosphate, adenosine‐5′‐diphosphate, l‐alanine, L‐tryptophan and L‐serine. The differential metabolites were visualized using a volcano plot (Figure 6A) and the representative differential metabolites and their related pathways were shown in Table 5.

**FIGURE 6:**
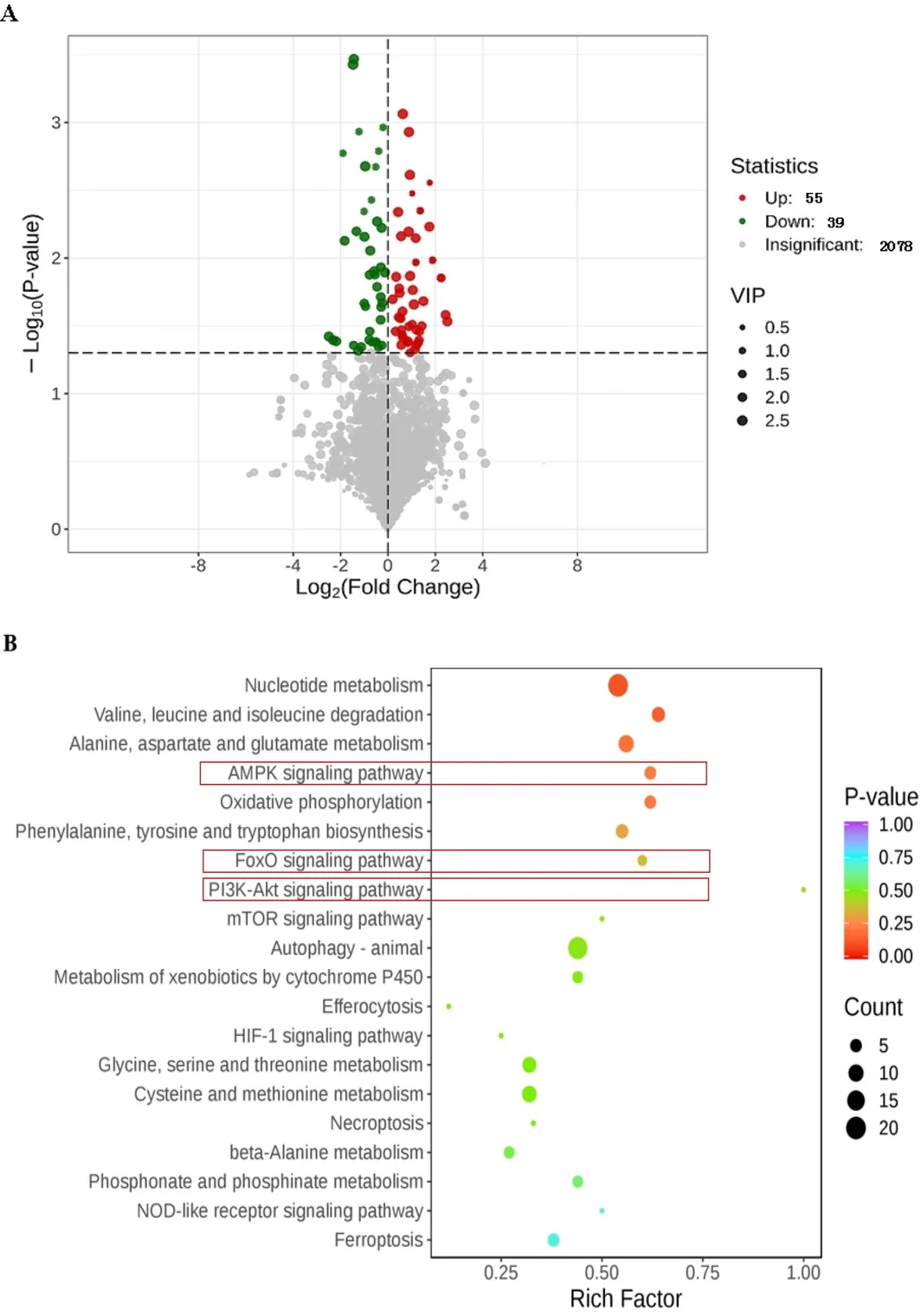
LC–MS/MS screening of differential metabolites and metabolomic pathways. (A) Volcano plot of differential metabolites. (B) KEGG analysis of pathway classification based on differential metabolite.

**TABLE 5:** Representative differential metabolites.

Index	Metabolites	Type	Correlative pathways
MW0103478	Adenosine monophosphate	Up	PI3k/Akt signalling pathway
MW0103469	Adenosine‐5′‐diphosphate	Down	FoxO signalling pathway
MW0105531	l‐Alanine	Up	Alanine, aspartate and glutamate metabolism
MW0105888	Asparagine	Up	Alanine, aspartate and glutamate metabolism
MW0106988	Glutamine	Down	Alanine, aspartate and glutamate metabolism
MW0168838	Betaine aldehyde	Up	Glycine, serine and threonine metabolism
MW0193946	L‐Tryptophan	Up	Glycine, serine and threonine metabolism
MW0109669	L‐Serine	Up	Glycine, serine and threonine metabolism
MW0169880	Succinic acid	Down	Oxidative phosphorylation
MW0106767	gamma‐Glutamylcysteine	Down	Ferroptosis

Furthermore, differential metabolites underwent pathway enrichment analysis. As shown in Figure 6B, KEGG analysis exhibited that multiple signalling/metabolic pathways, involving the PI3k/Akt signalling pathway, FoxO signalling pathway, AMPK signalling pathway (the top three signalling pathways), oxidative phosphorylation and multiple amino acid metabolic pathways, etc., were involved in the regulation of MLCD treatment in AGS cells.

### 3.7 Verification of MLCD‐Associated Targets in Gastric Cancer

Previous network pharmacology results have confirmed that multiple targets, such as TP53 and AKT1 and multiple pathways, including the PI3k/Akt signalling pathway, are involved in regulating MLCD against gastric cancer [21]. In this study, the metabolomics results highlight the top three roles of the AMPK pathway, FoxO signalling pathway and PI3k/Akt pathway. Therefore, we verified the expression of genes and proteins in the related pathways of MLCD anti‐gastric cancer using qRT‐PCR and Western blotting. Results showed that, in contrast to the control group, the mRNA and protein levels of P21, P53, LKB1 and AMPK increased with the increasing MLCD concentration (Figure 7).

**FIGURE 7:**
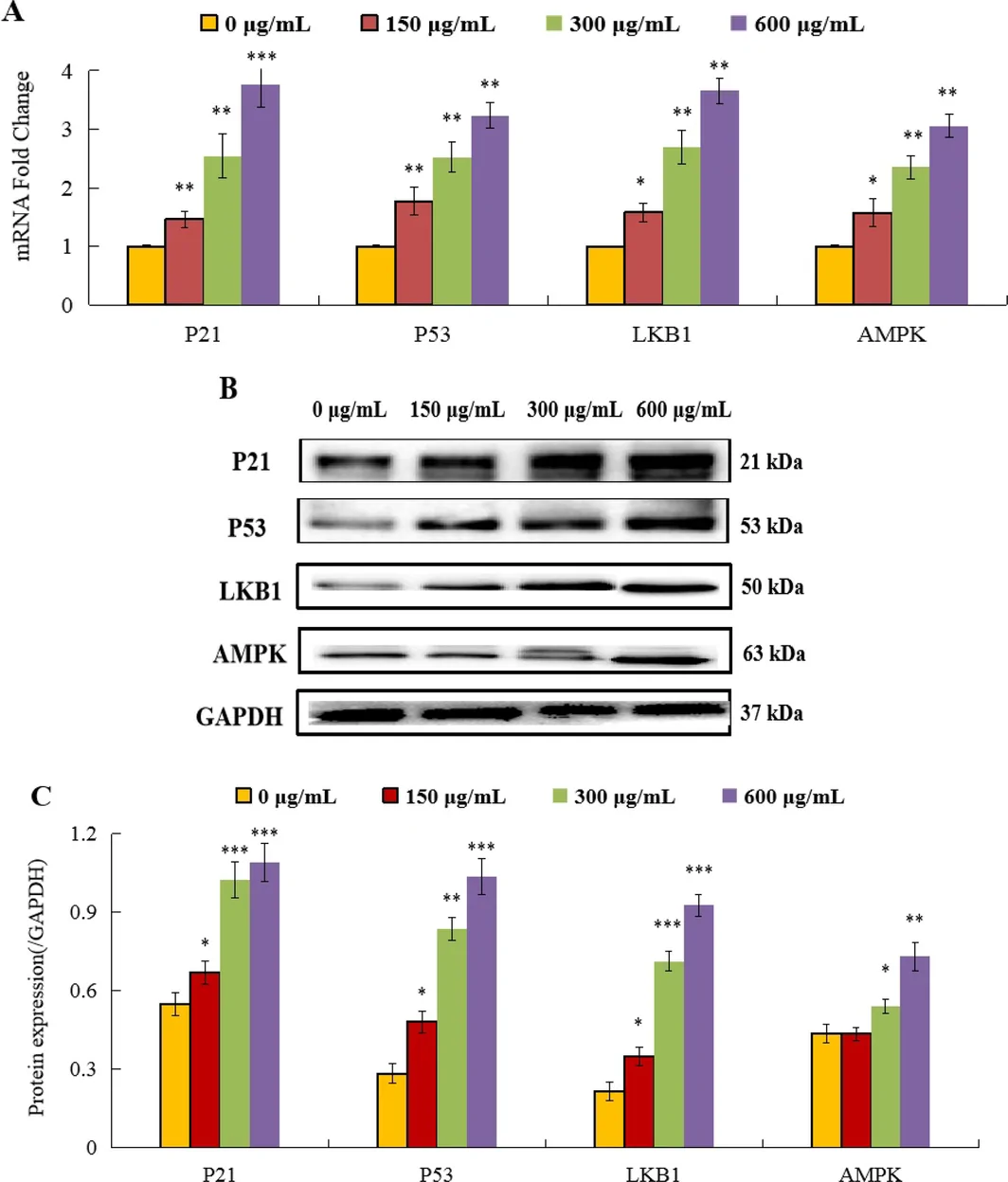
Verification of MLCD anti‐gastric cancer‐related targets. (A) The expression results of related genes were shown in AGS cells exposed to MLCD (0, 150, 300 and 600 μg/mL) for 48 h. A histogram showing the differences between groups. (B) The expression levels of related proteins were shown in AGS cells exposed to MLCD (0, 150, 300 and 600 μg/mL) for 48 h. (C) A histogram showing the differences between groups. All data are presented as the means ± SD from independent triplicate experiments. *p < 0.05, **p < 0.01, ***p < 0.001, compared with the control (0 μM) group.

### 3.8 Influence of MLCD on the Proliferation and Apoptosis in AGS Cells via the PI3k/Akt Pathway

The comprehensive metabolomics results and previous network pharmacology analyses, along with molecular docking of the main active ingredients with the AKT target, all emphasise the regulatory role of the PI3k/Akt pathway [21]. Hence, in this study, to confirm whether the PI3k/Akt pathway is involved in the regulation of MLCD on the proliferation and apoptosis in AGS cells, the CCK‐8 test was examined to assess the proliferative effect of MLCD or MLCD combined with LY294002. The results exhibited that MLCD prominently restrained the proliferation of AGS cells. Moreover, LY294002 combined with MLCD can increase the effects of MLCD on cellular proliferation (Figure 8A). Next, we evaluated the apoptotic rate via flow cytometer and the tendency of results coincided with the results of the CCK‐8 test (Figure 8B,C). Then, the expression levels of PI3k/Akt transduction pathway‐relevant factor were assessed via a qRT‐PCR test and a Western blotting test. All data confirmed that the expression level of the Bim gene was enhanced and the expression level of the FoxO3a gene was reduced, while an even greater degree of enhancement or reduction was surveyed for MLCD combined with LY294002 (Figure 8D). Besides, LY294002 prominently increased the MLCD‐suppressed phosphorylated protein of Akt and FoxO3a (Figure 8E). Further, as shown in Figure 8F, compared with the control group, the nuclear translocation of FoxO3a protein in AGS cells treated with MLCD (300 μg/mL) was observed under the microscope; the change of nuclear translocation was significantly enhanced when MLCD was combined with LY294002. Thus, these results suggest that the MLCD‐suppressed viability and ‐induced apoptosis in AGS cells may be related to the PI3k/Akt transduction pathway.

**FIGURE 8:**
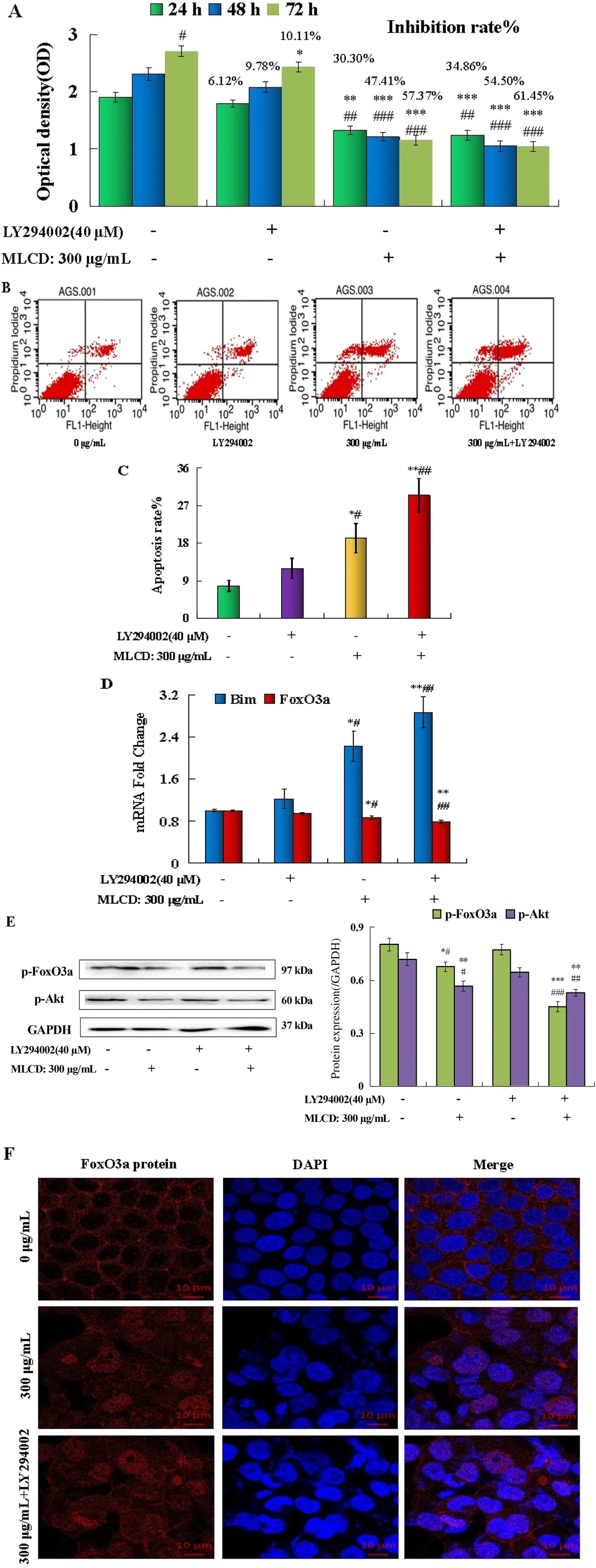
The PI3k/Akt transduction pathway is involved in inhibiting the proliferation and inducing apoptosis of AGS cells treated with MLCD or MLCD combined with LY294002. (A) CCK‐8 assay to assess proliferation. (B) Flow cytometry to analyse the apoptotic rate. (C) Quantitative results of apoptotic cells. (D) Expression levels of Bim and FoxO3a genes were detected via a qRT‐PCR test. (E) Expression levels of p‐Akt and p‐FoxO3a proteins were detected via a Western blotting test. (F) A representation of nuclear translocation. All data are presented as the means ± SD from independent triplicate experiments. *p < 0.05, **p < 0.01, ***p < 0.001, compared with the control (0 μM) group. ^#^ *p* < 0.05, ^##^ *p* < 0.01, ^###^ *p* < 0.001, compared with the LY294002 group.

## 4 Discussion

Our group's clinical practice discovered that the compound prescription of MLCD has good efficacy in improving gastrointestinal symptoms and prolonging life in patients with gastric cancer and has a certain potency in suppressing proliferation, avoiding metastasis and expediting cellular death in patients with gastric cancer. However, this still requires sufficient laboratory data to confirm.

In this study, the results of CCK‐8 tests and BrdU staining demonstrated that MLCD could inhibit proliferation in AGS cells. Morphological observation confirmed that MLCD caused cells to exhibit significant apoptotic characteristics and DNA gel electrophoresis and apoptotic rate analysis demonstrated that MLCD induced apoptosis in AGS cells. The results of gene and protein analysis discovered that MLCD could decrease the expression levels of the Bcl‐2 gene/protein and increase the expression levels of Bim, Bax, Fas and FasL genes/proteins, which were consistent with the reported literature.

Moreover, previous ingredient analysis of MLCD, combined with network pharmacology, confirmed that MLCD treatment of gastric carcinoma involved multi‐target (TP53, AKT1, HSP90AA and JUN, etc.) and multi‐pathway (PI3k/Akt pathway is the most significant) regulation. Meanwhile, molecular docking of the main active component with the AKT target also confirmed the involvement of the PI3k/Akt pathway [21]. In this study, the metabolomics results exhibited that MLCD could execute an anti‐gastric carcinoma effect via multiple signalling/metabolic pathways, including the PI3k/Akt signalling pathway, FoxO signalling pathway and AMPK signalling pathway, etc. Chinese herbs demonstrate anti‐tumour properties with multiple pathways and multiple targets. Based on previous results and the metabolomics results of this study, further verification of the targets and pathways related to apoptosis is warranted.

The P53/P21 pathway is one of the most important pathways mediating apoptosis. The P53 protein can bind to P21 by forming a homodimer, thereby regulating the cell cycle and the process of apoptosis [22]. It has been reported that P53 is involved in the response to various extracellular stimuli, thereby activating the apoptotic pathways related to mitochondria and death receptors, leading to apoptosis [23]. P53 can activate its downstream target gene P21. Weakened expression of the P21 protein may lead to a loss of effective monitoring of cell proliferation, increase the malignant phenotype of tumour cells and promote tumour initiation and progression [24]. In this study, gene and protein results discovered that MLCD could increase the expression levels of P53 and P21 genes/proteins.

The AMPK signalling pathway is a key energy sensor in both normal cells and cancer cells, playing a significant role in perceiving cellular energy availability. It also induces metabolic adaptation pathways to ensure cell survival. Once activated, it increases the catabolic production of ATP and inhibits ATP‐consumption biosynthetic processes [25]. Liver kinase B1 (LKB1), a tumour suppressor, functions as a primary upstream activator of AMPK and plays a pivotal role in cell growth and energy metabolism. It regulates cell structure, apoptosis and energy metabolic homeostasis by modulating a variety of downstream kinases [26]. Multiple studies have demonstrated that the LKB1/AMPK signalling pathway is involved in regulating the proliferation and apoptosis of cancer cells [27, 28, 29, 30]. In this study, gene and protein analyses discovered that MLCD could increase the expression levels of LKB1 and AMPK genes/proteins.

Reported studies indicate that cell proliferation and apoptosis are regulated by various signal transduction pathways. Among these, the PI3k/Akt pathway is a pivotal intracellular transduction pathway, playing a regulatory role in cell growth, survival and apoptosis [31]. LY294002, a specific inhibitor of PI3k pathways, can suppress the activation of Akt and its downstream factors. Furthermore, Bim, a pro‐apoptotic protein in the mitochondrial apoptotic pathway, is also a vital downstream factor of FoxO3a, which itself is a crucial downstream factor of the PI3k/Akt pathway; Bim significantly influences apoptosis in tumour initiation and progression [32]. Additionally, inhibition of the PI3k/Akt transduction pathway by applying LY294002 may lead to the translocation of FoxO3a protein from the cytoplasm into the nucleus [33]. The nuclear translocation of endogenous FoxO3a protein is considered a significant hallmark of apoptosis [34]. In this study, the results confirmed that pharmacological blockade by LY294002 could more effectively inhibit proliferation, induce apoptosis, influence the expression levels of PI3k/Akt transduction pathway‐relevant factors and enhance nuclear translocation of FoxO3a protein in AGS cells treated with MLCD, which was consistent with the reported literature.

In summary, the present results of our research show that MLCD can inhibit proliferation and induce apoptosis and that it may involve multiple pathways to exert anti‐gastric cancer effects. The study provides valuable information for MLCD in the therapy of gastric cancer. We believe that these results will help clarify its clinical application value and further consolidate its role as a potentially effective alternative medicine for the treatment of gastric cancer.

This research has some limitations. Primarily, this work revealed that MLCD can inhibit the proliferation and induce apoptosis in gastric cancer cells. However, whether there are other pharmacological effects, such as inhibiting invasion and metastasis or triggering autophagy still needs further exploration. Besides, we herein carried out metabonomics combining with the previous network pharmacology to confirm that MLCD can induce apoptosis by regulating the PI3k/Akt pathway. However, the mechanisms may need to be further demonstrated through genetic knockdown/overexpression studies or rescue experiments.

## Author Contributions

**Guoshan Shi:** methodology, writing – original draft, funding acquisition. **Liwei Ma:** funding acquisition, writing – original draft, conceptualization, project administration. **Hua Rong:** methodology. **Xu Li:** methodology, software. **Liping Wang:** methodology. **Shiyu Ni:** methodology, data curation. **Zhe Chen:** funding acquisition, writing – original draft, conceptualization, project administration.

## Funding

This study was sponsored by Science and Technology Project of Guizhou Province (Qiankehe Foundation MS [2026] 660), Qiqihar Academy of Medical Sciences (QMSI2024M‐01), Basic research business expenses of Heilongjiang Province Education Department (2025‐KYYWF‐ZR0985), Heilongjiang Provincial Postdoctoral Startup Project (LBH‐QY24005).

## Conflicts of Interest

The authors declare no conflicts of interest.

## Data Availability

Data and material will be provided on reasonable request.
